# A batch correction method for liquid chromatography–mass spectrometry data that does not depend on quality control samples

**DOI:** 10.1007/s11306-016-0972-2

**Published:** 2016-02-18

**Authors:** Martin Rusilowicz, Michael Dickinson, Adrian Charlton, Simon O’Keefe, Julie Wilson

**Affiliations:** 1 York Centre for Complex Systems Analysis, University of York, YO10 5GE, York UK; 2 Department of Computer Science, University of York, York, YO10 5DD UK; 3 Fera Science Ltd, York, YO41 1LZ UK; 4 Departments of Mathematics and Chemistry, University of York, York, YO10 5DD UK

**Keywords:** LC–MS, Mass spectrometry, Metabolomics, Quality control, Batch correction, QC correction

## Abstract

The need for reproducible and comparable results is of increasing importance in non-targeted metabolomic studies, especially when differences between experimental groups are small. Liquid chromatography–mass spectrometry spectra are often acquired batch-wise so that necessary calibrations and cleaning of the instrument can take place. However this may introduce further sources of variation, such as differences in the conditions under which the acquisition of individual batches is performed. Quality control (QC) samples are frequently employed as a means of both judging and correcting this variation. Here we show that the use of QC samples can lead to problems. The non-linearity of the response can result in substantial differences between the recorded intensities of the QCs and experimental samples, making the required adjustment difficult to predict. Furthermore, changes in the response profile between one QC interspersion and the next cannot be accounted for and QC based correction can actually exacerbate the problems by introducing artificial differences. “Background correction” methods utilise all experimental samples to estimate the variation over time rather than relying on the QC samples alone. We compare non-QC correction methods with standard QC correction and demonstrate their success in reducing differences between replicate samples and their potential to highlight differences between experimental groups previously hidden by instrumental variation.

## Introduction

Non-targeted metabolomic studies seek to analyse as wide a range of metabolites as possible. The use of liquid chromatography-mass spectrometry (LC–MS) for this purpose has found a wide range of applications, including drug discovery (Korfmacher 2005), disease biomarker discovery (Lu et al. 2008), pesticide (Zhang et al. 2011) and herbicide (Shalaby et al. 1992) analysis in agriculture, wastewater analysis (Kostich et al. 2014) and the discovery of novel metabolites (Nakabayashi and Saito 2013). LC–MS however suffers from lower reproducibility in comparison to other analytical techniques such as NMR spectroscopy (Gürdeniz et al. 2013; Rusilowicz 2014). Many non-targeted approaches focus on qualitative results, such as biomarker discovery, and the need for reproducible and comparable results is imperative, especially when differences between experimental groups are small. A number of factors can cause differences in LC–MS response profiles between acquisitions. Many of these relate to chromatographic aspects, such as retention time drift or changes in peak shape (Lai et al. 2009), but changes in the response of the mass spectrometer can also be seen (Ohlsson and Wallmark 1999). Most notable are the changes occurring during the acquisition of a multi-sample experiment due to the gradual contamination of the LC column. Whilst effective cleaning, conditioning and calibration of the instruments can mitigate these problems to a degree, consecutive analysis of large numbers of samples has been shown to present increasingly unacceptable variation (Zelena et al. 2009). Samples are therefore often run in batches, interspersed with the relevant cleaning and conditioning events. However, this can lead to other sources of technical variation, such as differences in the operating conditions under which the acquisitions of the individual batches are performed. The randomisation of sample order is essential as any correlation between experimental groups and batch would clearly be problematic.

Further sources of variation may be introduced in the early stages of data analysis. Although advances in methods of spectral alignment can reduce the effects of retention time drift and changes in peak shape, such methods do not always provide a complete solution in non-targeted studies involving thousands of potential metabolites. Spectral misalignment prior to the peak-picking stage can result in the classic problems seen in spectral binning, with differences between spectra being due to misaligned peaks rather than true changes in intensity.

A widely implemented solution to these problems is the inclusion of quality control (QC) samples into the study. During data acquisition the experimental samples are interspersed with a set of identical QC samples, providing a fixed reference point from which any instrumental variation can be tracked and later accounted for. The QC samples should contain the same metabolites as are under scrutiny in the study, being either a mixture of known laboratory grade analytes, or a pooled sample from the experiment itself. The former allows easier identification and quantitative analysis, whilst the latter allows as wide a range of metabolites as is attainable to be evaluated and is naturally more suited for non-targeted analysis. Should insufficient experimental samples be available for pooled samples, biologically similar samples may also provide reasonable QC data (Dunn et al. 2011; Van Der Kloet et al. 2009).

At the very least QCs can be used to gauge the reliability of the measurements for the individual metabolites. For example, in a GC–MS (gas chromatography-mass spectrometry) study, Begley et al. (2009) only accept individual metabolites where the relative standard deviation (RSD) of the QCs is less than 30 %. In another study involving DIMS (Direct Infusion Mass Spectrometry), Kirwan et al. (2013) use a limit of 20 % RSD with the additional criterion that the distribution of the QC samples be similar to that of the experimental ones. Other criteria have been proposed, for example that QC values should lie within 15 % of their mean (Begley et al. 2009; U.S. Department of Health and Human Services 2001).

However, since many sources of variation pertinent to the sample metabolites also apply to the QC metabolites, the function of the QC samples can be extended to correct for variation, rather than just quantify it. To do this a correction factor must be determined, for each metabolite and sample. Van Der Kloet et al. (2009) list several methods to achieve this, although the general form of the correction follows Eq. 1:

1 $$ {\text{X}^\prime}_{\text{p,b,i}} = X_{\text{p,b,i}} \frac{{R_{p} }}{{C_{p,b,i} }} $$

Here *X*_*p,b,i*_ is the intensity of peak *p* for sample *i* within batch *b*, prior to correction and *X*′_*p,b,i*_ is the corrected value. *C*_*p,b,i*_ represents the correction factor and *R*_*p*_ represents a rescaling factor which allows the relative intensity of the peak to be maintained. We refer to the set of correction factors*, C,* for a particular peak as the *trend* for that peak.

The simplest correction is to divide a peak within a sample by the average intensity recorded for that peak in the QC samples in the same batch as the sample, so that

2 $$ C_{p,b,i} = A_{p,b} = \mathop {average}\limits_{j\,\text{in}\,\text{Q}(\text{b})} \left( {X_{p,b,j} } \right) $$

Here *Q(b)* represents the QC samples in batch *b*, and *average* represents the averaging measure, which may be either the mean or the median. As the mean is more sensitive, its use may provide benefits when the number of observations is small, whereas the median offers a more robust measure, useful in cases where experimental outliers may affect the mean.

In (Van Der Kloet et al. 2009) the peak is rescaled to the average QC value for the first batch, hence the rescaling factor is $R_{p}   {\text{ = A}}_{p,1}$, whilst in (McKenzie 2013) it is suggested that the average peak intensity across all samples and batches be used and thus $R_{p}   {\text{ = A}}_{{p,1..N_{b} }}$ where *N*_*b*_ is the number of batches. Since changes in instrumental drift can be observed over time, per batch linear regression allows a degree of within-batch dynamics to be accounted for. A linear regression of QCs provides the correction factors:

3 $$ C_{\text{p,b,i}} = \beta_{b} {\text{i + }}\alpha_{b} $$

where *α*_*b*_ and *β*_*b*_ are the regression coefficients for batch *b.* Here, the integer *i*, relates to the *i*th sample for which data were acquired. Other, more advanced regression models including linear smoothers have also been used (Eilers 2003; Van Der Kloet et al. 2009). Dunn et al. (2011) apply the LOESS (LOcally WEighted Scatter-plot Smoother) algorithm to generate the trend-line for the QC samples in a method they term QC-RLSC (QC robust LOESS signal correction). LOESS is advantageous in that the data is modelled by a set of local polynomials, which avoids the constraint that the data follow any one global model and is less sensitive to errant data points (Cleveland 1979). The method requires optimisation of a smoothing parameter α.

Whilst QCs have been shown to provide an effective method for monitoring and correcting drift there has also been some success involving non-QC correction methods. It has been demonstrated that replicate measurements can be used to track experimental drift in lieu of periodic QC samples in a study involving ICP-OES (Inductively Coupled Plasma Atomic Emission Spectroscopy) (Salit and Turk 1998). This naturally allows more time to be dedicated to real sample analysis. The use of QC samples from pooled replicates has also been questioned because of observed inconsistencies between samples and pooled QCs (Ranjbar et al. 2012).

Checking the performance of any model can however be difficult, and it has been recognised that each dataset should be considered individually in order to determine which methods should be applied (Ranjbar et al. 2012). Kirwan et al. (2013) demonstrate success using a variation of the QC-RLSC that substitutes LOESS with a smoothing spline. Here the authors use RSD of technical replicates to determine the algorithm’s effectiveness, as did Ranjbar et al. (2012). Other methods have been proposed which avoid the need for technical replicates. Where QC samples are only used to determine variation, rather than correct for it, the total distance between the QC samples, or the RSD of the QC samples, can be used as a measure of instrumental variation. The distance between QC samples in principal component analysis (PCA) has been used to justify the idea that instrumental variation is not significant enough to be of concern (Gika et al. 2008). The predictive accuracy of partial least squares discriminant analysis (PLS-DA) on experimental groups has also been utilised to determine the effectiveness of correction (Prakash and Wei 2011). One-way repeated measures ANOVA has been used to calculate unexplained variation to determine the number of peaks for which the variance is reduced on the QCs (Ranjbar et al. 2012).

Here we explore data that is not amenable to QC correction due to the nature of the drift. The effects and performance of QC and non-QC correction methods are contrasted using these data. Previous studies have focussed on reducing batch or acquisition order differences, using the RSD of replicate samples as a method of gauging correction performance. Since we form the trends used to correct the data from experimental samples in addition to the QC samples, use of this measure could result in real differences between data points being erroneously removed. PLS classification has also been used as a measure of performance, however changes in the data that do not affect the classification rate cannot be detected. Here two evaluation methods are employed, both of which provide a metric of performance on a continuous scale. In addition to the mean RSD to measure the similarity of biological replicates we use PCA-MANOVA, a combination of Principal Components Analysis (PCA) and Multivariate Analysis of Variance (MANOVA), as a second measure of performance.

PCA is one of the most widely used multivariate techniques for exploratory analysis (Worley and Powers 2013). In PCA the coordinate system is rotated so that the first principal component (PC1) corresponds to the direction of maximum variance in the data with subsequent components (PC2, PC3, etc.) corresponding to progressively less variance. Data reduction is achieved by considering just the first few components accounting for most of the variance, and therefore most information, in the data. As an unsupervised method, PCA is commonly exploited in metabolomics studies to highlight experimental differences (Katajamaa et al. 2007; Rusilowicz 2014).

ANOVA (analysis of variance) can be considered a generalisation of the *t* test, allowing multiple groups to be considered. MANOVA is a multivariate extension of ANOVA that allows for multiple independent variables.

PCA-MANOVA therefore allows us to ascertain whether experimental conditions or LC–MS batch order are major sources of variation in our datasets and subsequently whether our improved “background correction” method facilitates a more robust determination of biological trends in our datasets.

## Materials and methods

### Experimental procedure

#### Sample collection and preparation

*Medicago truncatula*, a model legume, was subjected to individual biotic and abiotic stresses, and a combination thereof. A total of 150 plants were grown comprising four experimental groups as follows:

- C—Control group
- D—Abiotic stress group—subject to drought
- F—Biotic stress group—infected with the pathogen *Fusarium oxysporum*
- B—Dual stress group—subject to both drought and infection with *Fusarium*

Plants were planted in 350 ml pots containing a 3:1 mixture of perlite to sand by volume. Plants were grown in a greenhouse at a temperature of 28 °C and humidity was maintained using a fog system. *Fusarium* inoculation was carried out by watering the plants with 50 ml of *Fusarium* inoculate. Drought plants were subject to a 40 % drought stress by weight of water, a proportion determined to be effective from a previous pilot study.

Three plants (biological replicates) were harvested from each experimental group at daily intervals for 12 days. For the *C* and *F* groups 78 plants were harvested from days 1 to 12, whilst for D and B harvesting commenced 1 day later, from days 2 to 12 (72 plants), to allow uniform drying of the growth medium. Each plant was removed carefully from its substrate/gauze to minimise damage to the roots. The plant was shaken and the roots gently washed to remove any bound substrate. Roots were carefully dried before both leaves (*L*) and roots (*R*) were cut directly into beakers of liquid nitrogen. Only healthy mature leaves were cut whilst dead or very young leaves were discarded. After freezing, both leaves and roots were recovered from the nitrogen and stored in aluminium foil before freeze-drying for approximately 48 h. Lyophilised samples were then stored and transported for metabolomic analysis at room temperature.

Prior to analysis each dried sample was initially ground carefully into a fine powder using a pestle and mortar to preserve as much material as possible. Five mg ± 1 mg of ground sample was accurately weighed into a labelled 2 ml Eppendorf tube. To 5 mg of sample, 1 ml of extraction solvent (1:1 (v/v) methanol:water) was added. Metabolites were extracted into the solvent by shaking for 30 min. The solid material was then removed by centrifugation at 14,000 rpm for 10 min and the supernatant liquid split into two 400 µl aliquots, of which one was used for LC-HRMS (Liquid chromatography-high resolution mass spectrometry) analysis. The supernatant to be analysed by LC-HRMS was diluted fourfold using methanol: water 1:1.

In addition to the samples, an in-house reference was extracted daily as a QC measure. As the amount of material available from experimental samples was very low, the material for the QC samples was sourced from a homogenised mixture of control samples collected from a previous experiment following a similar design. This allowed the metabolites likely to be present in the experimental samples to be included in the QC samples without requiring the use of the limited experimental material in order to create the QCs.

#### LC-HRMS parameters

One hundred and forty nine leaf (*L*) and 148 root (*R*) samples were ultimately analysed—the number being slightly lower than anticipated (2 × 150) due to plants not attaining sufficient size for analysis or plant death. Extractions were subject to both positive (+) and negative (–) mode LC–MS, giving a total of four datasets (*L*+, *L*−, *R*+, *R−*). LC–MS analysis was conducted in seven batches to which the samples were assigned randomly to ensure that no particular batch was dominated by any particular experimental group or age-range.

The chromatography column used was an ACE 3Q 150 × 3 mm, 3 µm (Advanced Chromatography Technologies, Aberdeen, UK.). Mobile phases were 0.1 % formic acid in water (mobile phase A, MPA) and 0.1 % formic acid in acetonitrile (mobile phase B, MPB). The gradient elution applied was 100 % MPA for 5 min before increasing to 100 % MPB over 15 min. This was held for 10 min before reverting back to 100 % MPA and held for 2 min. Injection volume was 10 µl using a full loop injection, flow rate was 0.4 ml/min and column temperature was 25 °C.

The MS used was a Thermo Exactive (Thermo Fisher Scientific, MA, USA.) set at 50,000 resolution FWHM (full width at half maximum) (at 200 m/z) with an acquisition speed of 2 Hz. The column was conditioned before sample analysis using 15 QC injections and then QCs were inserted between every 6 experimental samples.

#### Data pre-processing

The raw LC–MS data were pre-processed using Progenesis QI (Nonlinear Dynamics, Newcastle Upon Tyne, UK). The software retention time aligned all MS spectra before applying deconvolution and peak picking algorithms providing a matrix of potential metabolites for each observation in a dataset. The potential metabolites were initially annotated by accurate mass *m/z* (between 80 and 1000) and retention time (between 1 and 30 min) of their corresponding peak. In reality some of these peaks may be due to erroneous peak detection or several peaks may represent the same compound. However, for brevity each peak will be referred to as a “metabolite” throughout. Table 1 shows the number of observations with the number of metabolites recorded for each dataset.

**Table 1:** The number of observations and metabolites (variables) for each of the four datasets

Leaf (L)		Root (R)	
184 observations (149 exp. +35 QC)		182 observations (148 exp. +34 QC)	
1239 L− metabolites	1681 L+ metabolites	4292 R− metabolites	4813 R+ metabolites

## Data analysis

It can be necessary to discard certain data points, for instance to remove noise peaks which present no useful information. Variables were removed from the dataset where the median of the QC values was zero (i.e. when 50 % or more of the QCs fail to show a value) to ensure that an accurate trend could be obtained. Similarly, when determining the trend using non-QC techniques, variables for which the median of all values was zero were removed. All data analyses were carried out in R (R Development Core Team).

### Assessment of performance

Performance was assessed using the mean RSD across all metabolites and replicates. For simplicity only replicate sets containing at least three observations were used, and values approaching zero (identified by at least one of the three or more values being zero in the original data, or containing all zeroes in the corrected data) were discounted. RSDs were calculated using the equation for the RSD of a subset (Rodbard 1974):

4 $$ RSD = \frac{\sigma }{{\overline{\overline{x}} }} $$

where *σ* is the standard deviation of the three replicates and $\overline{\overline{x}}$ is the grand mean for the metabolite. Our RSDs were calculated from the sets of biological replicates from plants exposed to the same experimental conditions for the same timepoints. It should be noted that in comparison to technical replicates, some differences are still to be expected, even if a perfect batch correction were to be performed, due to natural biological variation between the samples.

A combination of PCA and MANOVA was also used to judge the correction in terms of group separation. Data were mean centred and variables scaled to unit variance (divided by the standard deviation of the variable) prior to PCA to prevent metabolites with larger intensities dominating the scores.

MANOVA was used to provide an *F* statistic which shows the between group to within group variance ratio:

5 $$ F = \frac{variance\; between\; groups }{variance \;within \;groups} $$

Comparison of the F value with the appropriate F distribution gives a *p*-value for the significance of any difference between experimental groups. We used MANOVA on the PCA scores (coordinates of the rotated variables) for the first two principal components to quantify differences between experimental groups. This allowed the most apparent variations in the data to be considered in the MANOVA test. With an ideal correction the highest source of variation should be due to experimental groups rather than batch differences.

The groups considered in each test set are:

- Control and drought groups
- Drought and dual-stress groups
- Grouping due to LC–MS batch

We compared the control and drought groups as differences were already apparent in the uncorrected data and these should be retained by any correction method applied. Initial analysis showed little difference between the drought and dual stress groups and a correction method that could reveal these differences would be advantageous.

### Correction methods

The correction procedure involved the determination of the correction factors *C*_*p,b,i*_ shown in Eq. 1. This process was split into three stages. In the first stage the observations used to calculate the trend were selected: this could be based solely on the QCs, sets of replicates, or on all observations. The second stage involved selecting the method to be used to calculate the trend and in the third stage the observations to which the correction was applied are selected, i.e. individual batches or the full dataset.

In this analysis, correction methods were tested using only the QCs, but also using all observations (including QCs) to generate the trend, which we refer to as background correction. Both methods were tested on batches individually (batch-wise), and with the full dataset considered as one.

### Trend functions

The different methods used to determine the trend in the second stage were as follows:

#### *Mean*

The trend is set to the average of the samples, as in Eq. 2.

#### *Linear regression*

The trend is modelled via a linear regression of the samples.

#### *Moving median*

The trend is generated from the data using a simple moving average for smoothing. We used the median as analysis revealed that the moving mean resulted in unfavourable responses to individual high or low values (including genuine experimental values and not just outliers). For the moving median the correction factor *C*_*i*_ is calculated as the median of a moving window:

6 $$ C_{p,b,i} = median\left( {X_{p,b,i - w} ..X_{p,b,i + w} } \right) $$

where the *X*_*p,b,i*_ values used in the calculation are as defined for Eq. 1 and *w* is the window width.

#### *Polynomial regression*

Polynomial regression allows the data to be modelled as a simple *n*^th^ degree polynomial and requires the degree of the polynomial *n* to be specified.

#### *Smoothing spline*

The smoothing spline method fits a set of intersecting polynomials to the data. The function is controlled by a smoothing parameter *λ*, with larger values of *λ* leading to smoother functions (Hastie 1990). The smooth.spline algorithm from the R package stats (Ripley et al.) was used to generate the smoothed spline.

#### *LOESS*

LOESS combines multiple regression models and has previously been used to determine the correction factors both on QCs and on the full data set for DI-MS and LC–MS data (Kirwan et al. 2013; Kultima et al. 2009). Like the smoothing spline, LOESS is also controlled by a smoothing parameter.

### Method parameters

Several methods used to account for non-linear drift require parameters to be optimised. The window width *w* for the moving median, the degree *n* of the polynomial and the neighbourhood α that determines the smoothing parameter in LOESS were optimised to give the lowest mean RSD for biological replicates. The optimised parameters are listed in Table 2. Note that the correction using the batch-wise polynomial performed best with a polynomial degree of 1, effectively making it a linear correction. The smoothing spline was calculated using the R function smooth.spline with the default parameter set, which optimises the parameter λ via generalised cross validation in order to best fit the curve to the data (Ripley et al.).

**Table 2:** Table showing parameter values optimised in terms of RSD of biological replicates

Method	Parameter	Value
LOESS	Neighbourhood (*α*)	0.45
Batchwise LOESS	Neighbourhood (*α*)	0.5
Moving median	Window width (*w*)	5
Batchwise moving median	Window width (*w*)	5
Polynomial	Degree (*n*)	6
Batchwise polynomial	Degree (*n*)	1

## Results and discussion

**Fig. 1:**
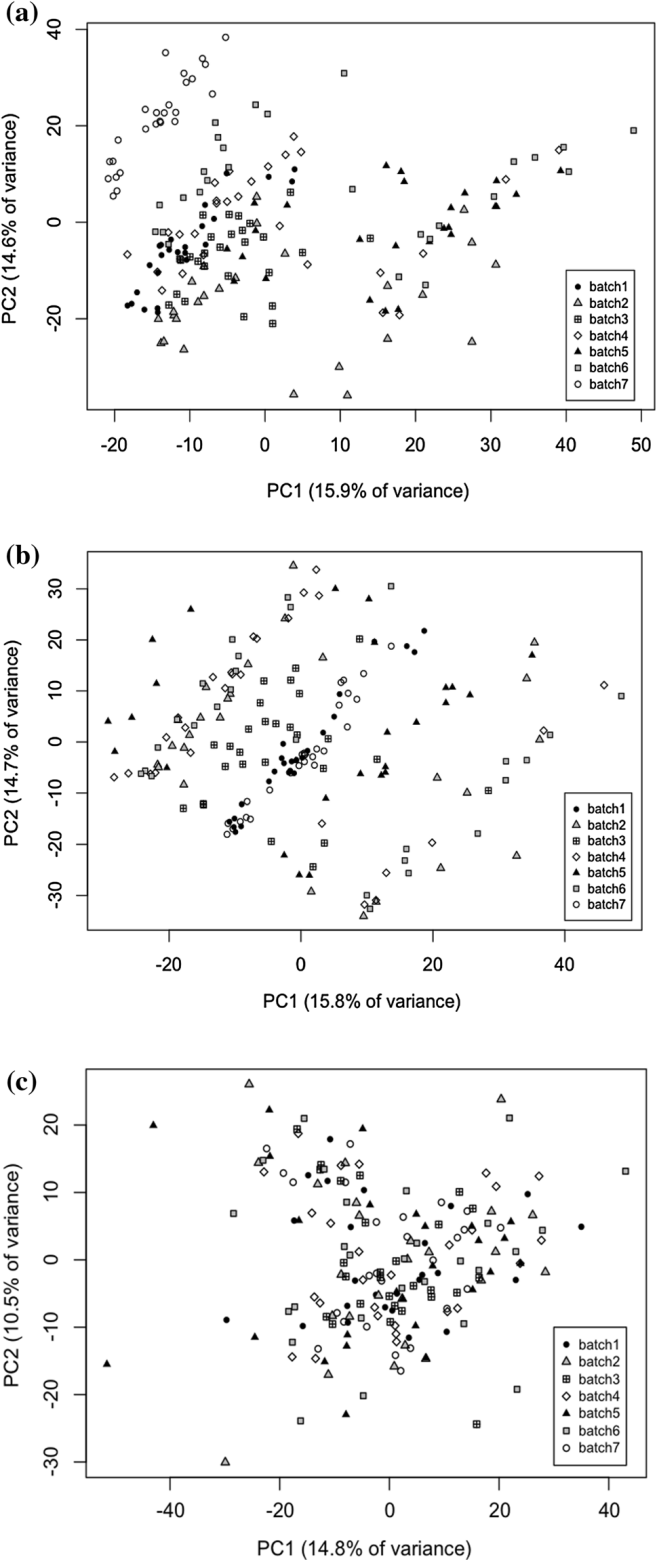
**a** The scores plot for the first two principal components of the scaled “L+” dataset showing batch differences as a major source of variation. **b** The scores plot after batch correction using the mean QC value, in which batch differences are made worse. **c** The scores plot after batch correction using the background correction method, in which batch differences are no longer apparent

**Fig. 2:**
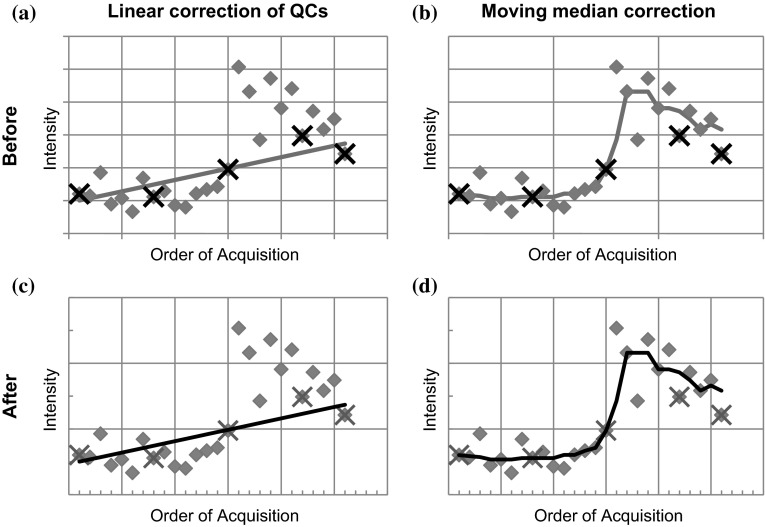
Plots showing how two methods of correction affect a metabolite and batch showing strong within-batch drift. Plots **a** and **b** show the values prior to correction, with the trend used for the two different correction methods shown by the bold line. **c** and **d** show the values post-correction, with the bold trend-line at 1.0. The linear correction (**c**) shows a notable pattern in the results when compared with the moving median correction in (**d**). *Diamonds* indicate observations, with QCs highlighted by crosses. The *line* indicates the correction factors forming the trend on which the corrections are based

For each dataset, it is clear from the Principal Components Analysis (PCA) of the scaled data that the majority of the variance is due to batch differences rather than experimental groups. Figure 1a shows the scores plot for the first two principal components for the L+ dataset. After batch correction using the traditional “mean of the QCs” method, PCA plots reveal that batch differences in the L−, R + and R– datasets are clearly reduced, with differences between the experimental groups becoming more apparent. However, this method was not able to correct for the batch differences in the L+ dataset as shown in Fig. 1b. It can be seen that several of the batches are “split” along the first principal component (PC1), with part of the batch having low scores for PC1 and the rest having higher scores. One of the implications of this is that the assumptions of standard statistical tests, such as t-tests or ANOVA may be invalid. Closer inspection of the L + dataset reveals that a large degree of *within*-*batch* drift can be observed for many metabolites, such as the example shown in Fig. 2a. Initial analyses of correction methods were also confounded by the presence of an outlier (*drought, day 6, replicate 3*), which was removed and the analysis repeated. Just as the median is more robust to outliers than the mean, robust PCA could potentially be employed to prevent the effects of outliers.

**Fig. 3:**
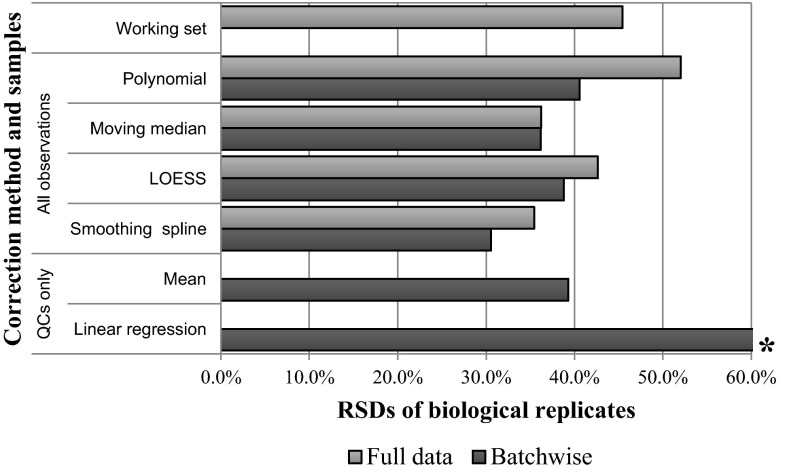
The mean relative standard deviation (RSD) using various correction methods. The working set represents the original data with an outlier observation and metabolites approaching the limit of detection removed, as described in the methods section. For each method the results are shown for the optimised parameters. *Asterisks* note that the results using the linear regression of the QCs have been truncated and the RSD is actually 193 %. Calculation of QC-only based techniques using the full dataset is not appropriate and is not shown. The working set is not corrected and hence only one value is displayed in the graph

At first sight, the use of linear regression modelling of the QCs in each batch to determine the trend appears to give improved results, as batch differences are no longer the greatest source of variance in the PCA. However batch differences are not eliminated and are now apparent along PC3. Furthermore, the method creates a number of outliers due to intensities being divided by very small numbers. This happens, for example, with metabolite #1283, which is responsible for the majority of variance along PC2 in unscaled PCA, and so is not restricted to peaks of low intensity. Patterns in the data when viewed in order of acquisition also remain, with sudden changes in the reported intensities within an individual batch that are not accounted for by a linear model. For example in batch 6, metabolite #1459 shows a drift in the experimental values different to that of the QCs (Fig. 2a). Such changes, which could have instrumental or analytical origins, lead to a poor fit of the linear regression model. The average RSD of the biological replicates, calculated across all variables and metabolites, shows that linear regression of the QCs leads to a huge increase in variation (Fig. 3). In fact the greatest source of variance seen in PCA is now due to artefacts introduced by the QC correction rather than to genuine differences between experimental groups.

Figure 3 shows that methods which use all observations reduce the batch variation more than methods based on the QCs alone. The comparatively poor performance of the QC based methods may be due to several factors:

- It can be problematic to determine an accurate trend due to the variation in the recorded intensities of the QCs.
- Since the QCs are placed intermittently they are unable to account for changes occurring at points between their placement.
- The number of QCs is low in comparison to the total number of observations, providing less information from which an accurate set of correction factors may be determined.

Background correction methods, i.e. techniques based on all observations (not just QCs), can follow the drift seen in the actual experimental samples of interest, allowing the correction of metabolites where the concentration is sufficiently different between QC and experimental samples. Figure 3 also shows that performing a background correction separately on each batch is more effective than ignoring batching and using all observations in a single background correction step. The average reduction in RSD achieved using batch-wise correction is 5.4 %. The difference is most apparent in polynomial correction, with the moving median being the least affected, possibly due to the moving median’s ability to rapidly track abrupt changes in the general flow of the data.

The best results, in terms of RSD between replicates, is achieved with the batch-wise smoothing spline with a 14.4 % reduction in RSD in comparison to the working set (the original data with variables classified as “noise” removed). The LOESS and the moving median correction methods both gave an improvement of ~9 % in comparison with the original data.

**Fig. 4:**
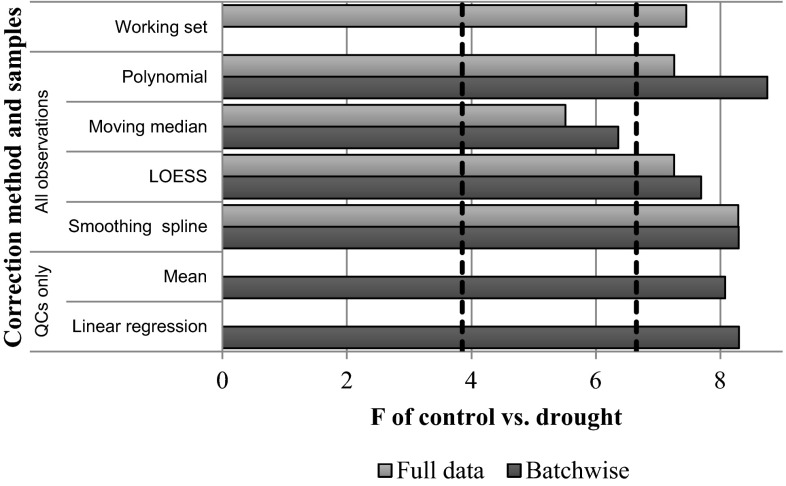
PCA-MANOVA results for the separation of *control* and *drought* experimental groups after batch correction using various techniques. A larger F statistic indicates a higher between-group to within-group variance ratio. Where applicable the techniques have been optimised to provide the lowest RSD across biological replicates. The *working set* represents the original data with metabolites approaching the limit of detection removed. The dotted lines show the critical F values of 3.85 for *p* = 0.05 and 6.65 for *p* = 0.01

The optimal parameters determined by RSD analysis are shown in Table 2. The correction methods were then evaluated using PCA-MANOVA. Figure 4 shows the PCA-MANOVA F statistics for control-drought discrimination are actually decreased by some batch correction methods in comparison to uncorrected data. In particular, the moving median, which gave good results in terms of RSD between replicates, gives a lower F statistic for the between group to within group variance ratio than for the working set. However the control-drought groups separate well prior to batch correction, with a *p* value of *0.001* for the F-test. The *p* value of 0.003 for the moving median shows the separation is still significant. The smoothing spline methods, which also showed good separation based on RSDs, show little difference in comparison to the uncorrected data, suggesting that, at the very least, we can apply these corrections without significantly damaging existing variations of interest.

**Fig. 5:**
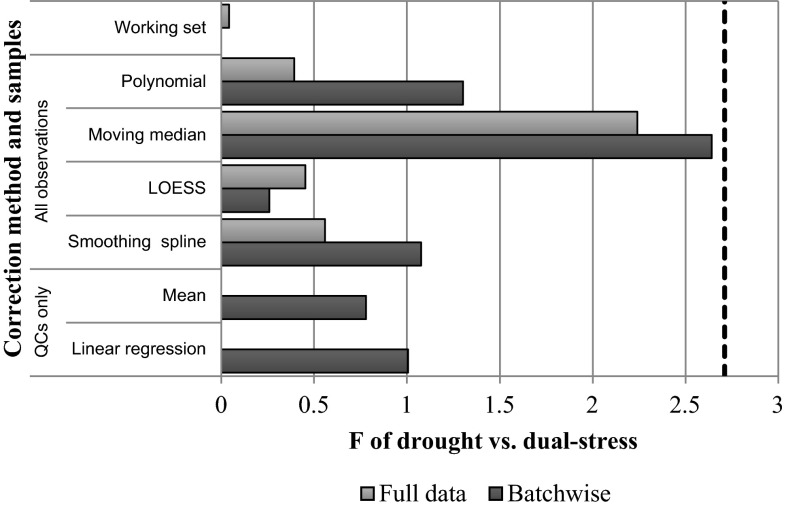
PCA-MANOVA results for the separation of *drought* and *dual*-*stress* experimental groups after batch correction using various techniques. A larger F statistic indicates a higher between-group to within-group variance ratio. Where applicable the techniques have been optimised to provide the lowest RSD across biological replicates. The *working set* represents the original data with metabolites approaching the limit of detection removed. The *dotted line* shows the critical F-value of 2.71 for *p* = 0.1

**Fig. 6:**
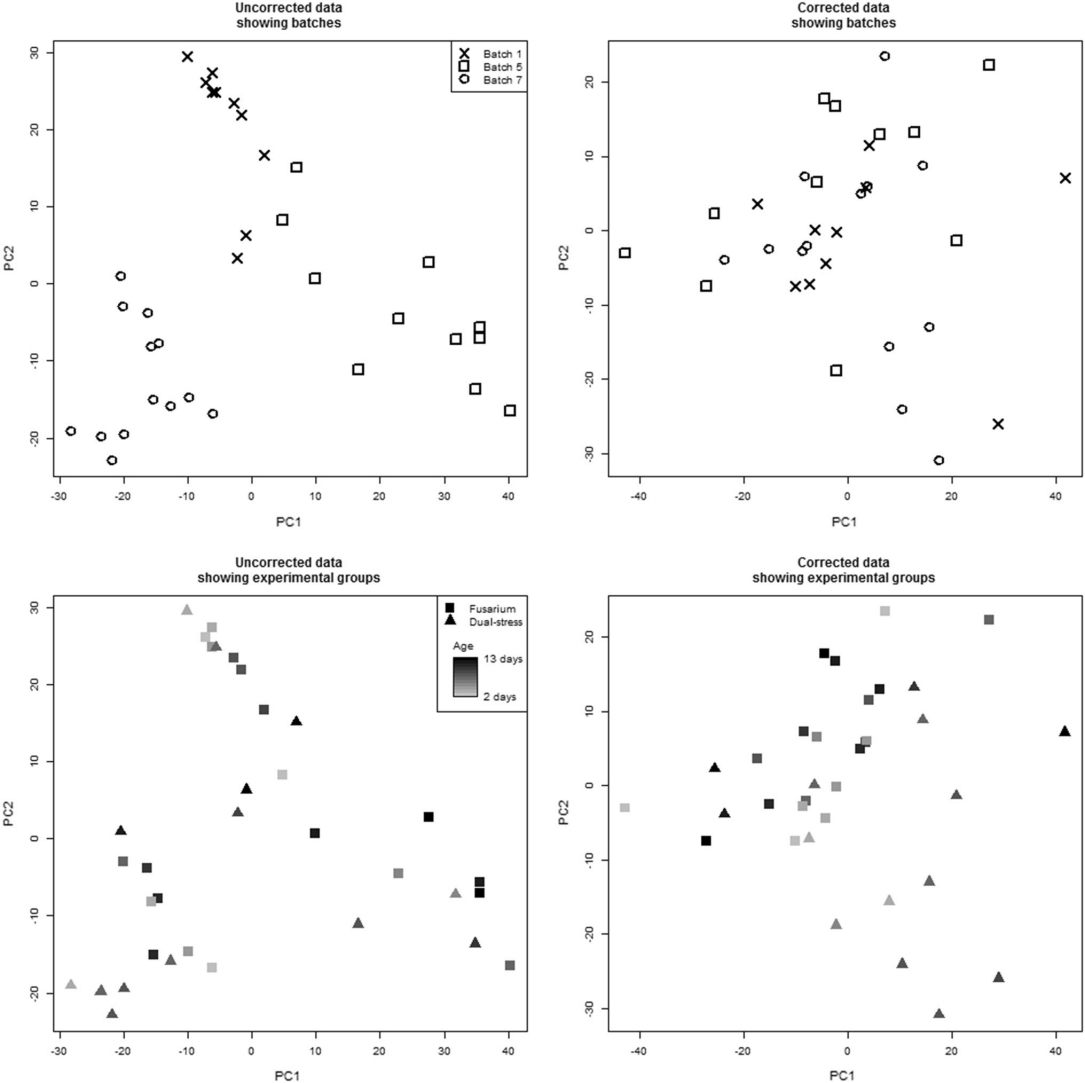
PCA scores plots of Fusarium and dual-stress samples for three batches, before and after background correction. The *top plots* show that obvious batch differences in uncorrected data are not evident after correction. The *lower plots* show the same data coloured according to experimental group with *darker colours* indicating samples from later in the time series

Figure 5 shows the PCA-MANOVA results for the drought and dual-stress groups. It can be seen that all correction methods give improved separation of experimental groups in comparison to uncorrected data. Interestingly, the moving median methods provide the best separation, performing considerably better than the smoothing spline methods. Figure 6 shows PCA scores plots for the Fusarium and dual-stress plants, before and after correction with the moving median, with just three batches shown for clarity. The increased separation of experimental groups can be seen.

PCA-MANOVA analysis of batch separation shows all correction methods provide a drastic reduction in batch differences, with only the uncorrected data having a significant F statistic. However, in some cases the F statistic may be reduced by the splitting of batches into two clusters, as shown in the PCA scores plot in Fig. 1. Since the different metrics of success yield different results this suggests that different correction techniques have their own merits and some may be more suited to certain situations than others.

In cases where QC samples do not truly represent the trends within batches, perhaps because insufficient samples are available, background correction using all samples (including QCs) provides a viable alternative. However, as QC samples should be identical and therefore most suitable for determining the correction factor, a hybrid method could potentially be developed in which more weight is given to QC samples.

## Concluding remarks

Where experimental drift occurs steadily throughout data collection, the overall trend may be identified using QC samples. However, jumps between batches require each batch to be treated individually and may result in insufficient QC samples to characterize the within-batch drift. In such cases improved correction may be achieved using a smoothed function of all observations within the batch to represent the trend. Background correction can be more effective than standard QC correction and does not necessarily require additional samples. Although the use of a batch-wise smoothing spline to represent the experimental drift was found to reduce the differences between biological replicates, all background correction methods evaluated provided better discrimination between experimental groups than uncorrected data. The use of a simple moving average not only gave good reduction in RSDs between replicates, but gave the highest between-group to within-group variance ratio for the drought and duel-stress groups, so that more complex smoothing methods may not be necessary. However, the moving median was less effective for the drought and control groups, where separation was already apparent in the uncorrected data. Just as scaling improves results in some situations and not others, different correction techniques may be more suited to some situations than others with no single method providing the optimal correction in all cases.
